# Factors Related to Sleep Disorders among Male Firefighters

**DOI:** 10.1186/2052-4374-26-11

**Published:** 2014-05-22

**Authors:** Dong-Kyun Lim, Ki-Ook Baek, In-Sung Chung, Mi-Young Lee

**Affiliations:** 1 Department of Occupational and Environmental Medicine, Dongsan Medical Center of Keimyung University, Daegu, Republic of Korea; 2 Department of Preventive Medicine, Keimyung University School of Medicine, Daegu, Republic of Korea

**Keywords:** Sleep disorders, Risk factors, Firefighters

## Abstract

**Objectives:**

The aim of this study was to investigate factors associated with sleep disorders in male firefighters working in a metropolitan city in South Korea.

**Methods:**

Self-administered questionnaires including the Nordic Musculoskeletal Questionnaire, Korean Occupational Stress Scale-Short Form, Psychosocial Well-Being Index-Short Form, Pittsburg Sleep Quality Index, and Beck-Depression Inventory-2 as well as surveys collecting socio-demographic characteristics and work-related factors were given to 730 male firefighters. After exclusion for missing data, 657 male firefighters were included, and logistic regression analysis adjusted for the work-related factors, psychosocial factors, and general risk factors were used to assess the relationship between sleep disorders and associated factors.

**Results:**

The prevalence of sleep disorders was 48.7%. Shift work (adjusted OR 1.58, 95% CI = 1.02-2.45), musculoskeletal symptoms (adjusted OR 2.89, 95% CI = 2.02-4.14), and depression (adjusted OR 7.04 95% CI = 4.03-12.30) were associated with sleep disorders.

**Conclusions:**

Musculoskeletal symptoms, shift work, and depression are associated with sleep disorders. Integrated health management is needed to promote good sleep quality among firefighters.

## Introduction

Sleep is essential for normal life and very important for health. Sleep disorders negatively influence the health of individuals sometimes causing gastrointestinal disorders, fatigue, and tension as well as negatively influence socio-economic potential through loss of productivity and can decrease concentration, which may lead to accidents and injuries [1,2]. As modern society changes, becomes more complicated, and grows, the prevalence of sleep disorders is increasing, and sleep disorders area rising social issue that may lead to economic losses not only for the individuals but also for the entire society. In South Korea, a telephone survey of 5,000 people 20–69 years of age found that 22.8% reported having a sleep disorder [3]. In addition, another study surveying normal adults reported that 31% suffered from sleep difficulties including difficulties falling asleep, waking up frequently, or waking up too early [4]. Moreover, among relevant studies conducted outside of South Korea, a majority of normal adults also reported suffering from a sleep disorder. For example, 10%–30% of normal adults in one previous study were found to be suffering from a sleep disorder [5-7].

Considering that firefighters in South Korea are national government officers who not only handle fire control/prevention but also perform rescue/first aid activities during disasters and other emergencies [8], exposure to physical and mental health problems as well as sleep disorders is highly likely. A recent study on sleep disorders among US firefighters reported that 59% of firefighters were suffering from a sleep disorder [9]. In Brazil, a study conducted in 2012 reported that 51% of firefighters were suffering from a sleep disorder [10]. In Iran, 69.9% of firefighters also had a sleep disorder [11]. Studies investigating sleep disorders among firefighters tend to indicate that the prevalence among firefighters is much higher than that of normal adults and sleep disorders among firefighters negatively affect health and the ability to perform daily activities [9-11].

Personal and occupational factors are related to the prevalence of sleep disorders. Personal factors include sex, age, living habits and environment, medical history, and any mental disorders [12,13], whereas occupational factors include incidents of occupational stress or traumatic events, employment type as well as various other factors that lead to mental and physical stress [14,15]. Firefighters are exposed to various harmful factors, urgent calls, and an irregular daily schedule [16], and this kind of occupational environment can lead to various health disorders such as physical and mental stress, which are believed to be related to the high rate of sleep disorders among firefighters. These health problems may be thought of as a personal problem, but should also be considered a social issue because firefighters serve as government officers in charge of social wellbeing. Therefore, a sustainable management program is necessary to resolve any health problems related to sleep disorders among firefighters.

We aimed to identify firefighters with sleep disorder sand analyze various factors that may affect sleep quality, such as socio-demographic, psychosocial, and occupational factors.

## Materials and methods

### Study population

Firefighters (n = 730, all males) from five fire stations in a metropolitan city in South Korea who visited a general hospital for an annual health examination between November 24, 2011 and December 22, 2011were surveyed. Among them, 73 subjects had missing responses leaving 657 firefighters as our final study population.

### Variables

After explaining the purpose of the study and receiving informed consent from all subjects, data were collected via standardized self-administered questionnaire. The questionnaire consisted of socio-demographic, occupational, and psychosocial characteristics as well as occupational stressors. Socio-demographic characteristics included age, marital status, alcohol intake, smoking, body mass index, and exercise. Occupational characteristics included years of service, shift work, departmental affiliation, and musculoskeletal symptoms. Psychosocial characteristics included sleep quality, depression, and psychosocial health, and occupational stressors were collected using 24 questions from the short form of the Korean Occupational Stress Scale.

Musculoskeletal symptoms were evaluated using the Korea Occupational Safety and Health Agency’s Guidelines on Occupational Harms Affecting the Musculoskeletal System (KOSHA code H-30-208) [17]. Those who experienced pain that persisted for more than a week during the previous year or experienced pain at least once a month (NIOSH Level 1) were classified accordingly.

The Korean version of the Pittsburgh Sleep Quality Index (PSQI) was used, which was developed by the University of Pittsburgh. The PSQI consists of seven factors and the sum of the points from all seven factors (0–21 points) determines sleep quality and the higher score the worse the sleep quality. We also applied the 6-point scale introduced by Buysse et al. [18] that classifies anyone with <6 points as the ‘good sleep group’ and those with ≥ 6 points as the ‘poor sleep group’.

For psychosocial health, the Psychosocial Well-Being Index Short Form [19], which summarizes the Psychosocial Well-Being Index and corrected for General health Questionnaire-60 for use in South Korea, was used to measure the level of psychosocial stress. The short form consists of 18 questions with a possible score between 0 and 54. Those with ≤8 points, 9–26 points, and 27 points were classified as the ‘healthy group’, ‘latent stress group’, and ‘stress group,’ respectively.

Depression was measured with the Beck Depression Inventory (BDI), which is the most widely used clinical survey in Korea, and is used to identify patients with depression. The BDI-2 is the modified version of the original BDI and is based on the new standards of depression diagnosis. It consists of 21 questions that apply a Likert-type scale of 0 through 3. The points from all 21 questions are added to obtain the total score (range 0–63 points). In the US, a score of 0–13 points, 14–19 points, 20–28, or 26–63 points from the BDI-2 is classified as ‘normal’, ‘mild depression’, ‘moderate depression’, or ‘severe depression’, respectively [20]. However, we adopted a more simple classification; those with scores <13 points or ≥13 points were classified as the ‘normal group’ or ‘depression group’, respectively.

The occupational stress factors were measured using the Korean Occupational Stress Scales Short Form that evaluates occupational stress factors across seven categories as follows: job demand, insufficient job control, interpersonal conflict, job insecurity, organizational system, lack of rewards, and occupational climate [21]. We identified the median scores of each of the seven categories and of the total score to classify subjects as either the ‘high stress group’ or ‘low stress group’ when their score was greater than the median score or less than the median score, respectively.

### Statistical analysis

The relativity between sleep quality (good vs. poor sleep) and age, years of service, or psychosocial health was analyzed using the linear–by-linear association, while the relativity between sleep quality and marital status, exercise, alcohol intake, smoking, obesity, musculoskeletal pain, shifts, employment, depression, occupational stress, and the total sum of all measured scores was analyzed using the chi-squared test. To identify factors that may influence sleep quality, factors with strong relativity among those with statistical significance in the univariate analysis were corrected for and applied as independent variables, and sleep quality was applied as the dependent variable in the logistic regression analysis. Model I included the socio-demographic characteristics as the independent variables, and model II applied the socio-demographic characteristics and musculoskeletal symptoms as the independent variables. Model III included the socio-demographic characteristics, musculoskeletal symptoms, occupational stress, and depression as the independent variables. Odds ratios (OR) and 95% confidence intervals (CI) were calculated. All data were analyzed using SPSS version 19.0 (IBM Corp., Armonk, NY, USA), and the significance level was set at 0.05.

## Results

### Socio-demographic characteristics

Within our total population of 657 subjects, the majority were aged between 30–40 years (79 subjects [12.0%] < 29 years, 221 subjects [33.6%] 30–39 years, 261 subjects [39.7%] 40–49 years, and 96 subjects [14.6%] ≥ 50 years). For years of service, 280 subjects (42.6%) had less than 10 years of service, 226 subjects (34.4%) had between 10 and 20 years, and 151 subjects (23%) had more than 20 years of service. In addition, 522 subjects (79.5%) were married, 325 subjects (49.5%) were exercising regularly more than 3 times/week, 395 subjects (59.8%) drank alcohol more than 2 times/month, 140 subjects (21.5%) were currently smoking, and 237 subjects (36.1%) were obese with a body mass index more than 25 kg/m^2^. Moreover, 514 subjects (78.2%) were shift workers, 499 subjects (76%) worked in the field, and 241 subjects (36.7%) had musculoskeletal pain (Table 1).

**Table 1:** General and psychosocial characteristics of the study population (n = 657)

**Variables**	**n**	**%**
Age (years)		
≤29	79	12.0
30 ~ 39	221	33.6
40 ~ 49	261	39.7
≥50	96	14.6
Tenure (years)		
<10	280	42.6
10 ~ 19	226	34.4
≥20	151	23.0
Marital status		
Unmarried	135	20.5
Married	522	79.5
Exercise		
No	332	50.5
Yes	325	49.5
Alcohol intake		
No	264	40.2
Yes	393	59.8
Smoking		
No	517	78.7
Yes	140	21.3
BMI^*^ (kg/m^2^)		
<25	420	63.9
≥25	237	36.1
Department		
Administrative job	158	24.0
Non-Administrative job	499	76.0
Musculoskeletal symptoms		
No	416	63.3
Yes	241s	36.7
Shift work		
No	143	21.8
Yes	514	78.2
Sleep quality		
Good	337	51.3
Poor	320	48.7
BDI-2^†^		
Normal range	536	81.6
Abnormal range	121	18.4
PWI-SF^‡^		
Healthy group	68	10.4
Latent stress group	482	73.4
Stress group	107	16.2

^*^Body mass index.

^†^Beck Depression Inventory-II.

^‡^Psychosocial Well-Being Index-Short Form.

There were 320 subjects (48.7%) with poor sleep quality and 121 subjects (18.4%) with depression. In addition, 68 subjects (10.4%) had normal levels of psychosocial stress, 482 subjects (73.4%) were in the latent stress group, and 107 subjects (16.2%) were in the stress group (Table 1). In terms of occupational stress, the mean scores ± standard deviations for job demand, insufficient job control, interpersonal conflict, job security, organizational system, lack of rewards, and occupational climatewere43.5 ± 15.1, 48.6 ± 12.0, 35.8 ± 10.9, 30.1 ± 16.7, 43.2 ± 12.8, 41.0 ± 12.5, and 36.7 ± 14.6, respectively. For the total score of occupational stress, the mean ± standard deviation was 39.9 ± 8.8 (Table 2).

**Table 2:** Mean (standard deviation) scores from the Korean Occupational Stress Scale (KOSS)

**Categories in the KOSS**	**Firefighter**	Reference^*^
		**(Q**_**25**_**-Q**_**75**_**)**^**†**^
Job demand	43.5 (15.1)	50.1 (41.7-58.4)
Insufficient job control	48.6 (12.0)	50.1 (41.7-66.7)
Interpersonal conflict	35.8 (10.9)	33.4 (33.3-44.5)
Job insecurity	30.1 (16.7)	50.1 (33.4-66.7)
Organizational system	43.2 (12.8)	50.1 (41.7-66.7)
Lack of rewards	41.0 (12.5)	55.6 (33.4-66.7)
Occupational climate	36.7 (14.6)	41.7 (33.4-50.1)
Total	39.9 (8.8)	48.5 (42.5-54.8)

^*^National Study for Development and Standardization of Occupational Stress

^†^Quartiles.

### Univariate analysis of risk factors

For the relativity between sleep quality and the socio-demographic characteristics or occupational characteristics, the ratio of poor sleep significantly decreased with age (p = 0.003) as we found 41 subjects (51.9%) younger than 29, 125 subjects (56.6%) in their 30s, 118 subjects (45.2%) in their 40s, and 36 subjects (37.5%) in their. For years of service, the ratio of poor sleep also significantly decreased with longer years of service (p = 0.007) with 147 subjects (52.5%) with less than 10 years of service, 116 subjects (51.3%) with 10 to 20 years, and 57 subjects (37.7%) with more than 20 years of service. In terms of alcohol intake, the ratio of poor sleep was significantly higher among alcohol drinkers (p = 0.045) with 204 alcohol drinkers (51.9%) and 116 non-drinkers (43.9%) in the poor sleep group. In addition, the ratio of poor sleep was higher among non-administrative workers; there were 259 non-administrative workers (51.9%) and 61 administrative workers (38.6%) who had poor sleep quality. For the relationship between musculoskeletal pain and sleep quality, those with musculoskeletal pain (n = 163; 67.6%) had a significantly higher sleep quality than did those with no musculoskeletal pain (n = 157; 37.7%) (p = <0.001). Moreover, shift workers (n = 265; 51.6%) were significantly more likely to have poor sleep than their counterparts were (n = 55;38.5%) (p = 0.006). Considering the relativity between psychosocial characteristics and sleep quality, 10 subjects were in the healthy group (14.7%) based on their psychosocial stress score, 220 subjects were in the latent stress group (45.6%), and 90 subjects were in the stress group. These data indicate that the ratio of poor sleep increased with psychosocial stress (p < 0.001). Moreover, those with depression (n = 103; 85.1%) were significantly more likely to have poor sleep than the normal group (n = 217; 40.5%) was (p < 0.001) (Table 3). The relativity between occupational stress and sleep quality indicated that those with higher than the median scores for job demand (p = 0.001), insufficient job control (p = 0.001), job insecurity (p = 0.030), organizational system (p < 0.001), lack of rewards (p < 0.001), occupational climate (p < 0.001), and total job stress (p < 0.001) were significantly more likely to have poor sleep than their counterparts were (Table 4).

**Table 3:** Correlation of sleep quality with general and psychosocial factors

	**Sleep quality**		
**Variables**	**Good**	**Poor**	p-value^*^
	**n (%)**	**n (%)**	
Age (years)			
≤29	38 (48.1)	41 (51.9)	0.003
30 ~ 39	96 (43.4)	125 (56.6)	
40 ~ 49	143 (54.8)	118 (45.2)	
≥50	60 (62.5)	36 (37.5)	
Tenure (years)			
<10	133 (47.5)	147 (52.5)	0.007
10 ~ 19	110 (48.7)	116 (51.3)	
≥20	94 (62.3)	57 (37.7)	
Marital status			
Unmarried	66 (48.9)	69 (51.1)	0.531
Married	271 (51.9)	251 (48.1)	
Exercise			
No	168 (50.6)	164 (51.3)	0.720
Yes	169 (52.0)	156 (48.0)	
Alcohol intake			
No	148 (56.1)	116 (43.9)	0.045
Yes	189 (48.1)	204 (51.9)	
Smoking			
No	275 (53.2)	242 (46.8)	0.061
Yes	62 (44.3)	78 (55.7)	
BMI (kg/m^2^)^†^			
<25	217 (51.7)	203 (48.3)	0.799
≥25	120 (50.6)	117 (49.4)	
Department			
Administrative job	97 (61.4)	61 (38.6)	0.004
Non-Administrative job	240 (48.1)	259 (51.9)	
Musculoskeletal symptoms			
No	259 (62.3)	157 (37.7)	<0.001
Yes	78 (32.4)	163 (67.6)	
Shift work			
No	88 (61.5)	55 (38.5)	0.006
Yes	249 (48.4)	265 (51.6)	
BDI-2^‡^			
Normal range	319 (59.5)	217 (40.5)	<0.001
Abnormal range	18 (14.9)	103 (85.1)	
PWI-SF^§^			
Healthy group	58 (85.3)	10 (14.7)	<0.001
Latent stress group	262 (15.9)	220 (45.6)	
Stress group	17 (15.9)	90 (84.1)	

^*^Calculated using theχ^2^-test and linear-by-linear association.

^‡^Beck Depression Inventory-II.

^†^Body mass index.

^§^Psychosocial Well-Being Index-Short Form.

**Table 4:** Correlation between Korean Occupational Stress Scale (KOSS) score and sleep quality

	**Sleep quality**		
**Categories in the KOSS**	**Good**	**Poor**	p-value^*^
	**n (%)**	**n (%)**	
Job demand			
Low	280 (54.9)	230 (45.1)	0.001
High	57 (38.8)	90 (61.2)	
Insufficient job control			
Low	240 (56.2)	187 (43.8)	0.001
High	97 (42.2)	133 (57.8)	
Interpersonal conflict			
Low	274 (52.4)	249 (47.6)	0.267
High	63 (47.0)	71 (53.0)	
Job insecurity			
Low	327 (52.2)	299 (47.8)	0.030
High	10 (32.3)	21 (67.7)	
Organizational system			
Low	288 (55.3)	233 (44.7)	<0.001
High	49 (36.0)	87 (66.2)	
Lack of reward			
Low	224 (59.3)	154 (40.7)	<0.001
High	113 (40.5)	166 (59.5)	
Occupational climates			
Low	231(60.8)	149(39.2)	<0.001
High	106 (38.3)	171(61.7)	
Total			
Low	307 (54.6)	255 (45.4)	<0.001
High	30 (31.6)	65 (68.4)	

^*^Calculated using theχ^2^-test.

### Multivariate risk factor analysis

Among the factors that were significantly related with sleep quality in univariate analysis, we found the relationships between age and years of service, shifts works and department of service, and psychosocial stress and depression to have strong relativity. Therefore, these factors were considered independent variables and added to our three logistic regression models to analyze their relativity with sleep quality. Model I corrected for age, alcohol intake, and shift work, model II corrected for musculoskeletal symptoms and all of the factors from model I, and model III corrected for occupational stress and depression in addition to the factors from model II. In model I, shift work had an OR of 1.66 (95% CI = 1.12–2.47) for poor sleep. In model II, alcohol intake had an OR of 1.41 (95% CI = 1.01–1.97), shift work had an OR of 1.63 (95% CI = 1.08–2.46), and musculoskeletal pain had an OR of 3.37 (95% CI = 2.40–4.74). Last, in model III, shift work had an OR of 1.58 (95% CI = 1.02–2.45) and musculoskeletal pain had an OR of 2.89 (95% CI = 2.02–4.14). In addition, depression was robustly associated with poor sleep with an OR of 7.04 (95% CI = 4.03–12.30) (Table 5).

**Table 5:** Logistic regression analysis of factors related to sleep quality

			**OR**^**§ **^**(95% CI**^ǁ^**)**			
**Variables**	Model I^*^		**Model II**^**†**^		**Model III**^**‡**^	
Age (years)						
≤29	1.00		1.00		1.00	
30 ~ 39	1.38	(0.82-2.34)	1.32	(0.77-2.29)	1.15	(0.63-2.01)
40 ~ 49	0.86	(0.52-1.44)	0.91	(0.53-1.55)	0.71	(0.41-1.24)
≥50	0.74	(0.39-1.40)	0.76	(0.40-1.47)	0.57	(0.28-1.14)
Alcohol intake						
No	1.00		1.00		1.00	
Yes	1.37	(0.99-1.87)	1.41	(1.01-1.97)	1.34	(0.94-1.92)
Shift work						
No	1.00		1.00		1.00	
Yes	1.66	(1.12-2.47)	1.63	(1.08-2.46)	1.58	(1.02-2.45)
Musculoskeletal symptoms						
No			1.00		1.00	
Yes			3.37	(2.40-4.74)	2.89	(2.02-4.14)
BDI-2^¶^						
Normal range					1.00	
Abnormal range					7.04	(4.03-12.30)
Total job stress**						
Low					1.00	
High					0.93	(0.93-2.72)

^*^Model I was adjusted for age, alcohol intake, and shift work. ^†^Model II was adjusted for model I and musculoskeletal symptoms.

^‡^Model III was adjusted for model II, depression, and total job stress. ^§^Odds ratio ^ǁ^confidential interval ^¶^Beck Depression Inventory-II.

^**^Sum of categories score/7.

## Discussion

We aimed to identify sleep disorders and its related factors within a population of male firefighters. In total, 320 out of 657 male firefighters (48.7%) had poor sleep quality, which is higher than the reported prevalence of sleep disorders in normal adults (10%-30%) [5-8]. In addition, in our multivariate logistic regression analysis, sleep quality was significantly related to shift work, depression, and musculoskeletal pain.

Previous studies have identified that shift work is related to various health problems such as cardiovascular diseases, metabolic syndrome, sleep disorders, mental diseases, digestive diseases, and cancer [22]. Some studies have reported that sleep disorders are strongly related to shift work. For example, one study found shift work to agitate the circadian rhythm and degrade sleep quality [23]. Another study reported that shift work causes physiological malfunctioning leading to sleep disorders, loss of appetite, and digestive problems [22]. There are also reports that workers in small manufacturing businesses and nurses are prone to suffer from sleep disorders [24,25]. Therefore, it is expected that firefighters may suffer from sleep disorders possibly due to their shift work and irregular schedules. We found that 78.2% of firefighters in our study were working in shifts and the shift workers had a higher ratio of sleep disorders than the non-shift workers did.

Depression is one of the psychiatric disorders with a high prevalence in both common people and working people groups [26]. Firefighters confront various occupational stressors that can be dangerous and urgent due to their working environment, the structure of their worksite, and interpersonal relationships. These stressors are known as risk factors leading to depression among firefighters [27], and occupational stress and symptoms of depression are strongly related to one another [28]. In addition, firefighters may become exposed to musculoskeletal disorders and the pain that results may also contribute to the development of depression [29]. Depression can cause various health problems and sleep disorders [30], which was an important factor in our study. One study reported that depression degrades sleep quality and may cause insomnia [31]. Moreover, depression and sleep disorders are risk factors for each other with mutual causality [32].

Psychosocial stress is significantly higher in groups that are tense or active at work [33]. Firefighters frequently experience tense moments at work because they cannot anticipate the critical conditions and always have to be ready for urgent calls. They also experience psychological stress in the field or when their co-workers are victims of accidents, and may undergo post-traumatic stress after working in severely damaged sites and seeing dead bodies or tragic injuries in the field [34]. These psychological stressors might explain the relationship with sleep disorders [5], and our study confirms these findings. However, psychosocial stress can cause other health problems such as depression and musculoskeletal symptoms [35-37]. As we previously mentioned, depression and musculoskeletal symptoms may be related to sleep disorders, and psychosocial stress may affect depression and musculoskeletal pain that might also indirectly influence sleep disorders.

The firefighters that work in the field can become overly stressed, perform repetitive and stressful movements in inappropriate positions, and overwork their muscles when using maximum strength at fire scenes or rescue scenes. In addition, they wear heavy personal protective gear and work in narrow, dangerous spaces that impose physical stress on their shoulders, lower back, and knees. Such a poor working environment increases the possibility of musculoskeletal symptoms [38], and psychological stress can have a combined affect on the occurrence of musculoskeletal symptoms [36,37]. The musculoskeletal symptoms caused by mental and physical stress can directly affect sleep quality through pain [6], but chronic musculoskeletal symptoms, as mentioned above, can increase depression symptoms [27] and indirectly affect sleep quality simultaneously.

Although not significantly related to sleep in the multivariate logistic regression analysis, age, alcohol intake, and occupational stress were found to be significantly related to sleep disorder in the univariate analysis. Age is a known risk factor of sleep disorders and preceding studies have reported that sleep disorders increase with age [39,40]. However, our results indicate that poor sleep tends to decrease with age. One explanation could be that those with longer years of service and higher positions have smaller workloads and a heightened ability to cope with stress as they age and experience less physical and mental stress. We found that the mean Psychosocial Well-Being Index score decreased with age when the score was stratified by age. Alcohol intake has been found to decrease REM sleep time and negatively influence total sleeping time [41]. However, a study in Japan reported that alcohol intake induces sleep [42]. Our study did not show a significant relativity between alcohol intake and sleep quality, but we did not classify alcohol intake into different levels of the exposure. Future studies should stratify alcohol intake across different levels to analyze the relativity between alcohol intake and sleeping more accurately. Several preceding studies have reported that occupational stress directly and indirectly influences sleep [42-44]. In our univariate analysis, we identified that all subscales of occupational stress, except for interpersonal conflict and the total score of occupational stress, had significant relativity with sleep. However, the multivariate logistic regression analysis did not reveal a significant relativity between the total score of occupational stress and sleep quality. Depression tends to be strongly correlated with occupational stress and may have confounded and/or weakened our analysis of the relativity between total job stress and sleep quality.

There were three main limitations of our study. First, our study was cross-sectional; therefore, the causality between sleep quality and its related factors cannot be confirmed. A future prospective study is needed to provide supplementary data. Second, other factors that may influence sleep quality, such as past medical history, noise in the living environment, and other living conditions were not comprehensively collected for data analysis. In addition, our subjects included a limited population of male firefighters; thus, our findings cannot be generalized to female firefighters or all firefighters. Finally, we used self-administered questionnaires that reflect the subjective judgment of our subjects and are prone to bias. Nevertheless, this study analyzed factors that affect sleep quality among firefighters in terms of their socio-demographic, occupational, psychosocial, and occupational stress factors. In addition, we used the PSQI, Psychosocial Well-Being Index Short Form, BDI-2, and Korean Occupational Stress Scale Short Form all of which are reliable and valid measures used to study sleep quality.

## Conclusion

In conclusion, firefighters may be exposed to a greater risk of sleep disorders than other groups of people. Moreover, sleep quality among firefighters is affected by various factors including physical, mental, occupational, and bio-environmental factors. These health problems affect not only their personal life but also society. Personal health problems can deteriorate their satisfaction and immersion in the workplace and create difficulties in the field. Therefore, greater attention should be paid to health problems and sleep disorders, which also affect health. To improve sleep quality among firefighters, their working environment and health conditions should be studied carefully. By doing so, effort can be made to improve poor working environments, analyze and assess the results of health diagnosis, and understand their relationship with the working environment and health problems to reduce the physical and psychological stress among firefighters. In addition, an integrated health control program that considers their physical and mental/psychological health should be developed in addition to a regular health control system for each risk factor. These measures should be applied to provide active health management for individual firefighters who suffer from sleep disorders.

## Competing interest

The authors declare that they have no competing interests.

## Authors’ contribution

DKL was responsible for data analysis, and for drafted this manuscript. MYL was responsible for the content of the manuscript, for study design, for data analysis, and for drafted this manuscript; KOB was responsible for the data collection; ISC was responsible for the data analysis and interpretation. All authors contributed to the drafting and revisions of the manuscript. All authors read and approved the final manuscript.

## References

[B1] Rajaratnam SMW Barger LK Lockley SW Shea SA Wang W Landrigan CP O’Brien CS Qadri S Sullivan JP Cade BE Epstein LJ White DP Czeisler CA Sleep disorders, health, and safety in police officers JAMA 2011 26 23 2567 2578 10.1001/jama.2011.1851 22187276

[B2] Edéell-Gustafsson UM Kritz EI Bogren IK Self-reported sleep quality, strain and health in relation to perceived working conditions in females Scand J Caring Sci 2002 26 2 179 187 10.1046/j.1471-6712.2002.00078.x 12000672

[B3] Cho YW Shin WC Yun CH Hong SB Kim J Earley CJ Epidemiology of insomnia in korean adults: prevalence and associated factors J Clin Neurol 2009 26 20 23 10.3988/jcn.2009.5.1.20 19513329 PMC2686894

[B4] Choi SH Kim I Seo KY A study on the sleep patterns of the general adult population in Seoul J Korean Neuropsychiatr Assoc 1992 26 2 289 310 Korean

[B5] Simon GE VonKorff M Prevalence, burden, and treatment of insomnia in primary care Am J Psychiatry 1997 26 1417 1423 9326825 10.1176/ajp.154.10.1417

[B6] Ohayon MM Smirne S Prevalence and consequences of insomnia disorders in the general population of Italy Sleep Med 2002 26 115 120 10.1016/S1389-9457(01)00158-7 14592229

[B7] Morin CM LeBlanc M Daley M Gregoire JP Mérette C Epidemiology of insomnia: prevalence, self-help treatments, consultations, and determinants of help-seeking behaviors Sleep Med 2006 26 123 130 10.1016/j.sleep.2005.08.008 16459140

[B8] Kim SK Health hazards in firefighters Hanyang Medical Reviews 2010 26 4 296 304 10.7599/hmr.2010.30.4.296

[B9] Carey MG Al-Zaiti SS Dean GE Sessanna L Finnell DS Sleep problems, depression, substance use, social bonding, and quality of life in professional firefighters J Occup Environ Med 2011 26 928 933 10.1097/JOM.0b013e318225898f 21785370 PMC3486736

[B10] Viviam VB Leonardo FM Richard S Ronaldo RB Telmo MR Mental health conditions, individual and job characteristics and sleep disturbances among firefighters J health Psychology 2012 26 3 350 358 10.1177/1359105312443402 22517948

[B11] Mehrdad R Haghighi KS Esfahani AH Sleep quality of professional firefighters Int J Prev Med 2013 26 9 1095 1100 24130955 PMC3793495

[B12] Ford DE Kamerow DB Epidemiologic study of sleep disturbances and psychiatric disorders: an opportunity for prevention? JAMA 1989 26 1479 1484 10.1001/jama.1989.03430110069030 2769898

[B13] Marquié JC Foret J Sleep, age, and shift work experience J Sleep Res 1999 26 297 304 10646170 10.1046/j.1365-2869.1999.00170.x

[B14] Sohn JG Kim JH Kim HC Combined effects of individual background and job stress on the development of sleep problems in blue-collar workers Kosin University College of Medicine 2006 26 1 248 255 Korean

[B15] Cothereru C Beaurepaire C Payan C Cambou JP Rouilllon F Conso F Professional and medical outcomes for French train divers after “person under train” accidents: three years follow up study Occup Environ Med 2004 26 488 494 10.1136/oem.2003.007922 15150387 PMC1763648

[B16] Kim MH A study on the effectiveness and characterisitics of management system in fire administration 1996 Seoul: Master’s Dissertation, Korea University Korean

[B17] Korea Occupational Safety and Health Agency Guideline of harmful factors survey for musculoskeletal overloading works Available: http://www.kosha.or.kr/content.do?menuId=1751 [cited 13 December 2013]

[B18] Buysee DJ Reynolds CF Monk TH Berman SR Kupfer DJ The Pittsburgh sleep quality index: a new instrument for psychiatric practice and research Psychiatr Res 1989 26 2 193 213 10.1016/0165-1781(89)90047-4 2748771

[B19] Chang SJ Standardization of Collection and Measurement of Health Statistics Data 2000 Seoul: The Korean Society for Preventive Medicine 111 135 Korean

[B20] Beck AT Steer RA Brown GK Beck Depression Inventory (2nd Manual) 1996 San Antonio: The Psychological Corporation

[B21] Chang SJ Standardization of job Stress Measurement Scale for Korean Employee 2004 Incheon: Korea Occupational Safety and Health Agency 17 41 Korean

[B22] Kim YG Yoon DY Kim JI Chea CH Hong YS Yang CG Kim JM Jung KY Kim JY Effect of health on shiftwork: general and psychological health, sleep, stress, quality of life Kor J Occup Environ Med 2002 26 3 247 256 Korean

[B23] Tepas DI Carvalhais AB Sleep patterns of shift workers Occup Med 1990 26 2 199 208 2203155

[B24] Lee KS Lee DB Kwon LS Cho CY Depressive symptoms and their association with sleep quality, occupational stress and fatigue among small-scaled manufacturing male workers Korean J Occup Environ Med 2011 26 2 99 111

[B25] Lee JT Lee KJ Park JB Lee KW Jang KY The relations between shift work and sleep disturbance in a University Hospital Nurses Korean J Occup Environ Med 2007 26 3 223 230

[B26] Stewart WE Ricci JA Chee E Hahn SR Morganstein D Cost of lost productive work time among US workers with depression JAMA 2003 26 3135 3144 10.1001/jama.289.23.3135 12813119

[B27] Guidotti TL Human factors in firefighting: ergonomic-, cardiopulmonary-, and psychogenic stress-related issues Int Arch Occup Environ Health 1992 26 1 1 12 10.1007/BF00625945 1399009

[B28] Saijo Y Ueno T Hashimoto Y Job stress and depressive symptoms among Japanese fire fighters Am J Ind Med 2007 26 6 470 480 10.1002/ajim.20460 17471508

[B29] Rajala U Keina¨nen-Kiukaanniemi S Uusima¨ ki A Kivela¨ SL Musculoskeletal pains and depression in a middle-aged Finnish population Pain 1995 26 3 451 457 10.1016/0304-3959(94)00206-T 7478689

[B30] Morgan K Clarke D Risk factors for late-life insomnia in a representative general practice sample Br J Gen Pract 1997 26 166 169 9167321 PMC1312924

[B31] Nicolas A Dorey JM Charles E Clement JP Sleep and depression in elderly people Psychol Neuropsychiatr Vieil 2010 26 3 171 178 20739255 10.1684/pnv.2010.0224

[B32] Lustberg L III R Charles F Depression and insomnia; questions of cause and effect Sleep Medicine Review 1999 26 3 253 262 10.1053/smrv.1999.0075 12531168

[B33] Chang SJ Cha BS Koh SB Kang MG Koh SR Park JK Association between job characteristics and psychological distress of industrial workers J Prev Med Public Health 1997 26 129 144 Korean

[B34] Dudek B Koniarek J Relationship between sense of coherenceand post-traumatic stress disorder symptoms among firefighters Int J Occup Med Environ Health 2000 26 4 299 305 11276843

[B35] Nieuwenhuijsen K Bruinvels D Frings-Dresen M Psychosocial work environment and stress-related disorders, a systematic review Occup Med 2010 26 4 277 286 10.1093/occmed/kqq081 20511268

[B36] Leino P Magni G Depressive and distress symptoms as predictors of low back pain, neck-shoulder pain, and other musculoskeletal morbidity: a 10-year follow-up of metal industry employees Pain 1993 26 1 89 94 10.1016/0304-3959(93)90060-3 8316395

[B37] Bonger PM Winter CR Kompier MAJ Hidebrandt VH Psychosocial Factors at work and musculoskeletal disease Scand J Work Environ Health 1993 26 297 312 10.5271/sjweh.1470 8296178

[B38] Bos J Mol E Visser B Frings-Dresen M Risk of health complaints and disabilities among Dutch firefighters Int Arch Occup Environ Health 2004 26 6 373 382 15338222 10.1007/s00420-004-0537-y

[B39] Ohayon MM Carskadon MA Guilleminault C Vitiello MV Meta-analysis of quantitative sleep parameters from childhood to old age in healthy individuals: developing normative sleep values across the human lifespan Sleep 2004 26 1255 1273 15586779 10.1093/sleep/27.7.1255

[B40] Torsvall L Akerstedt T Gillberg M Age, sleep and irregular work hours. A field study with electroencephalographic recording, catecholamine exertion and self-rating Scand J Work Environ Health 1981 26 196 203 10.5271/sjweh.3112 20120585

[B41] Lobo LL Tufik S Effects of alcohol on sleep parameters of sleep-deprived healthy volunteers Sleep 1997 26 52 59 9130335 10.1093/sleep/20.1.52

[B42] Pelfrene E Vlerick P Kittle F Mak RP Kornitzer M De Backer G Psychosocial work environment and psychological well-being: assessment of the buffering effects in the job demand-control (-support) model in BELSTRESS Stress Health 2002 26 43 56 10.1002/smi.920

[B43] Nasermoaddeli A Sekine M Hamanishi S Kagamimori S Job strain and sleep quality in Japanese civil servants with special reference to sense of coherence J Occup Health 2002 26 337 342 10.1539/joh.44.337

[B44] Kim MO The Study of Risk Factors of Sleep Disorder Symptoms in Subway Drivers 2007 PhD thesis: Department of Medicine, Graduate School Public Health, Pusan National Univ Korean

